# The effect of a new developed synbiotic yogurt consumption on metabolic syndrome components, oxidative stress status, and some other cardiovascular disease risk factors in adults with metabolic syndrome: a study protocol for a randomized clinical trial

**DOI:** 10.1186/s40795-023-00723-y

**Published:** 2023-06-06

**Authors:** Mohammad-Amin Zolghadrpour, Farzad Karimpour, Mohammad-Reza Jowshan, Hossein Imani, Somayyeh Asghari

**Affiliations:** 1 Department of Clinical Nutrition, School of Nutritional Sciences and Dietetics, Tehran University of Medical Sciences, No#44, Hojjatdoust St., Naderi St., Keshavarz Blvd, Tehran, 141556117 Iran; 2 Social Determinants of Health Research Center, Yasuj University of Medical Science, Yasuj, Iran

**Keywords:** Synbiotic yogurt, Probiotic, Metabolic syndrome, Oxidative stress, Cardiovascular disease, Clinical trial

## Abstract

**Background:**

Metabolic syndrome is recognized as one of the most common global health issues, which may cause numerous side effects. Studies have shown the favorable effects of probiotic supplements on glycemic indices, lipid profiles, and oxidative stress status. However, the number of studies investigating the effects of food products containing probiotics and prebiotics on metabolic diseases is limited. Limited evidence also shows that products containing Lactobacillus plantarum could affect metabolic alterations in chronic diseases. No previous study evaluated the impact of synbiotic yogurt containing Lactobacillus plantarum on people with metabolic syndrome. Therefore, the current study aims to investigate the effect of the newly developed synbiotic yogurt containing Lactobacillus plantarum, Lactobacillus pentosus, and Chloromyces marcosianos yeast on the components of metabolic syndrome, oxidative stress status, and some other risk factors for cardiovascular diseases in adults with metabolic syndrome.

**Methods:**

In this study, 44 patients with metabolic syndrome will be randomly assigned to intervention and control groups in a randomized, double-blind, controlled clinical trial. Participants in the intervention group will consume 300 g of synbiotic yogurt daily, while those in the control group will consume 300 g of regular yogurt daily for 12 weeks. Anthropometric measurements, blood pressure, and biochemical parameters will be evaluated before and after the intervention.

**Discussion:**

The management of the metabolic syndrome presents significant clinical challenges. While probiotic supplementation for these individuals has been considered, the consumption of probiotic-rich foods has received considerably less attention.

**Trial registration number:**

Iranian Registry of Clinical Trials (IRCT20220426054667N1) (2022–05-18).

## Introduction

The Metabolic syndrome (MetS) is recognized as one of the most common global health issues [1]. Lifestyle modifications, such as improving eating habits and physical activity, are the main therapeutic strategies to manage MetS complications [2]. Numerous side effects, such as type 2 diabetes, cardiovascular disease, non-alcoholic fatty liver disease, polycystic ovary syndrome, several types of cancer, inflammatory bowel disease, and chronic kidney disease, are common in subjects with MetS [3]. Insulin resistance, hypertension, dyslipidemia, and visceral obesity are the components of MetS according to the National Cholesterol Education Program (NCEP) criteria [4].

Studies have shown that oxidative stress, inflammation, and insulin resistance are considered major factors in the pathophysiology of the MetS [5]. By inducing lipolysis in adipose tissue, insulin resistance causes a high quantity of free fatty acids to be released into the bloodstream, which increases the oxidation of free fatty acids and the generation of reactive oxygen species (ROS) [6]. The increase in ROS production leads to oxidative stress and a decrease in antioxidant defenses [7]. Oxidative stress results in the conversion of low-density lipoprotein (LDL) to oxidized low-density lipoprotein (ox-LDL), which has been shown to be increased in patients with MetS [8]. Ox-LDL exerts cytotoxic effects on vascular endothelial cells and is a significant contributor to atherosclerosis and endothelial dysfunction [9]. In addition, it causes the endothelial barrier to break down and reduces the bioavailability of nitric oxide (NO) [10]. In addition, individuals with MetS have been shown to have higher levels of malondialdehyde (MDA), a marker of lipid peroxidation in oxidative stress conditions [11]. Understanding the pathogenic processes that contribute to the development and progression of MetS, as well as modifying associated risk factors, are crucial to the management and treatment of the disease [12].

In recent years, the importance of the gut microbiome in the pathogenesis of metabolic disorders associated with MetS has been highlighted [13]. Dysbiosis or imbalance in the gut microbiome may contribute to the development of oxidative stress, inflammation, and other cardiometabolic risk factors [14]. The composition of the gut microbiome can be modified by a variety of methods. Consuming probiotics and prebiotics is one of these methods [15]. It has been shown that probiotics and synbiotics play a crucial role in controlling MetS risk factors such as dyslipidemia, insulin resistance, abdominal obesity, blood pressure, and atherosclerosis [16]. In addition, probiotics help to reduce oxidative stress by regulating ROS-producing enzymes, enhancing the activity of antioxidant enzymes, and eliminating oxidant compounds from the gut or preventing their production [17]. Furthermore, active peptides are produced by probiotic bacteria and can function as antioxidants [18].

The newly developed synbiotic yogurt that will be used in this study contains native strains of Lactobacillus plantarum, Lactobacillus pentosus, and Chloromyces marcosianos yeast [19]. In human studies, probiotic supplements containing Lactobacillus plantarum have been demonstrated to improve glycemic indices, lipid profiles, and oxidative stress [20]. Besides, limited evidence shows that products containing Lactobacillus plantarum could favorably affect metabolic and oxidative alterations in chronic diseases [21]. However, the number of studies investigating the effects of probiotic and prebiotic food products on metabolic diseases is limited. On the other hand, no studies have been conducted to evaluate the effect of synbiotic yogurt consumption containing Lactobacillus plantarum in people with MetS. In order to develop effective approaches for the prevention and management of the MetS complications, the current study aims to investigate the effect of the newly developed synbiotic yogurt containing Lactobacillus plantarum, Lactobacillus pentosus, and Chloromyces marcosianus on the components of MetS, oxidative stress status, and some other risk factors for cardiovascular diseases in adults with MetS.

## Material and methods

The study protocol has been approved by the ethics committee of Tehran University of Medical Sciences (ethics number: IR.TUMS.MEDICINE.REC.1401.080). Participants will be given a comprehensive explanation of the study's objectives, methods, benefits, and risks. All participants will also be informed that taking part in the research is completely voluntary, and they can quit at any time that they want. A written informed consent will be collected from every participant prior to the study's enrollment. The trial was registered at the Iranian Registry of Clinical Trials (www.irct.ir) with the number IRCT20220426054667N1.

### Participants

This study will be conducted as a parallel randomized clinical trial with an allocation ratio of 1:1. Patients with MetS aged 30 to 50 and body mass index (BMI) of 25 –35 kg/m^2^ will be recruited from health centers affiliated with Yasouj University of Medical Sciences, Yasouj, Iran. MetS will be confirmed based on the ATP III criteria when at least three of the following criteria are met: waist circumference (WC) > 102 cm in men and > 88 cm in women, triglyceride (TG) ≥ 150 mg/dl, high-density lipoprotein (HDL) ≤ 40 mg/dl in men and ≤ 50 mg/dl in women, blood pressure ≥ 130 to 85 mmHg, and fasting blood sugar (FBS) ≥ 100 mg/dl.

We will exclude those followed weight loss programs or weight changes of more than 10% of initial weight during the last six months; professional athletes or subjects with any changes in the intensity and quantity of physical activity in the previous four weeks; pregnant, lactating and postmenopausal women; those who have allergy to dairy products and probiotics; smokers and alcoholic beverage consumers; those who routinely consume probiotic or synbiotic yogurts; individuals with cardiovascular, lung, nervous system, kidney, liver, thyroid, and other endocrine diseases, diabetes, cancer, and eating disorders; taking drugs that affect appetite, body weight, and lipid metabolism, corticosteroids, oral contraceptives, antidepressants and antipsychotics, lipid lowering medications, antibiotics, antidiabetic and blood pressure medications (uncontrolled blood sugar and blood pressure); receiving probiotics and other dietary supplements in the last three months.

### Sample size calculation

Sample size was calculated using the following formula, with inputs including the mean and standard deviation of TG changes in the intervention (212.8 ± 21.1) and control (201.9 ± 19.6) groups obtained from the Cicero et al. study [21], 95% confidence, and 80% power of the test. The minimum sample size in each group was calculated to be 17 cases. However, after adjusting for possible dropouts, this number increased to 22 cases in each group.

### Study design

**Fig. 1:**
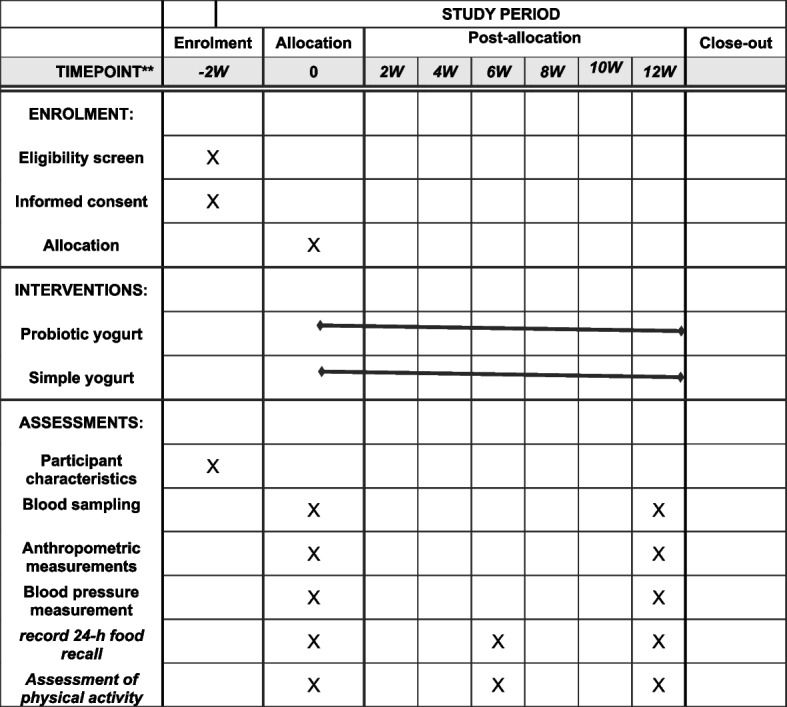
Template of content for the schedule of enrolment, interventions, and assessments

Prior to the primary intervention, all selected individuals will participate in a two-week "run-in" period during which relevant questionnaires are used to gather information regarding sociodemographic variables, the history of diseases, drug and supplement use, dietary intakes, and physical activity levels. At the end of the run-in period and before the start of the intervention, people will be randomly assigned to the intervention and control groups. A computerized randomization will be carried out using a block randomization procedure of size 2 and 4, stratified by gender (male or female), and BMI (25–30 or 30–35 kg/m^2^). Each patient will get an identity number, which will be input into the computer's randomization program together with the codes of patients who have the same age and BMI. The intervention or control groups will ultimately be randomly allocated to patients with the same conditions. Random allocation will be performed by a person unaffiliated with the study. Subjects in the intervention group will consume 300 g of synbiotic yogurt and those in the control group will consume 300 g of regular yogurt daily for 12 weeks (Fig. 1). Participants will be asked not to change their regular physical activity and usual dietary intakes during the study. They will also be asked not to use any other probiotic or synbiotic products while participating in the trial. The participants' dietary intake will be evaluated again at the end of the sixth week as well as at the end of the trial in order to evaluate the patients' nutritional intake during the study and to ensure yogurt consumption. Physical activity level will also be assessed during the sixth week and the last week. Blood samples (10 cc) will be obtained after a 12-h overnight fast at baseline and at week 12 for biochemical measurements (Table 1).

**Table 1:** Product delivery program and follow up participants

Visit time	Run in week -2(day-14)	meeting day (day 0)	Week 2 (day 14)	Week 4 (day 28)	Week 6 (day 42)	Week 8 (day 56)	Week 10 (day 70)	Week 12 (day 84)	Week 13 (day 91)
**Delivery of yogurt**		*	*	*	*	*	*	*	
**Anthropometric measurements**		*						*	
**Blood pressure measurement**		*						*	
**Blood sampling**		*						*	
**record 24-h food recall**	*	*			*			*	
**Assessment of physical activity**	*	*			*			*	
**End of intervention**								*	
**Evaluation of adverse events**		*	*	*	*	*	*	*	*

### The intervention

The synbiotic yogurt that will be used in this study contains strains of Lactobacillus plantarum, Lactobacillus pentosus (2˟10^8^ CFU), Chloromyces marcosianos yeast, and 3% of various natural plants (celery, shallot, chicory, and mint). The colony rate of both bacterial strains was reported as 2˟10^8^ by microbiological analysis; however, before the intervention, yogurt quality control will be carried out under the supervision of the Food and Drug Administration laboratory in Yasuj, Iran, to confirm the presence of this colony rate. The maximum degree of similarity will be observed in both yogurts, and there will be no labels on the containers to blind both the researchers and subjects to the type of the yogurt they received. In the case of a serious adverse event, patients are unmasked to ensure their safety. In these circumstances, unblinding is carried out using a set of operationalized guidelines by an unblinded medical assessor who has access to randomization while not actively participating in the trial. For each participant, the amount of yogurt required to consume during two weeks will be packed and delivered every two weeks. Participants will be asked to keep the yogurt bottles in the refrigerator. To remind them to consume yogurt, they will receive weekly calls and short messages. Compliance with the consumption of yogurt will be monitored by counting the number of returned unused and used yogurt containers every two weeks. Evaluating the consumption of the yogurts will also be done through food recalls.

### Anthropometric measurements

Anthropometric parameters, including weight, height, BMI, and WC, will be measured for all participants at baseline and at the end of the study. A digital scale with a 100-g precision will be used to measure the subject's weight while wearing light clothing and no shoes. The standing height of people will be measured using a standard height meter without shoes and with an accuracy of 0.5 cm. WC will be measured using a tape measure in the middle of the distance between the superiliac bone and the last rib with an accuracy of 0.5 cm. Then the BMI will be calculated by dividing the weight (kg) by the squared of the height (m).

### Assessment of dietary intakes and physical activity level

A 24-h dietary recall questionnaire will be used to assess the daily dietary intakes of the study subjects during two weekdays and one weekend day at baseline, at the end of the sixth week, and at the end of the trial. Nutritionist IV software will be used to determine participants' macronutrients and micronutrients intakes using the data obtained from food recalls.

Physical activity level will be evaluated at baseline, during the sixth week, and at the last week using the international physical activity questionnaire (IPAQ), which has been validated for the Iranian population [22]. Physical activity level in terms of MET-h/wk will be calculated according to the activity performed using the standard MET table and will be classified into three levels including low, moderate, and high intensity physical activity.

### Blood pressure measurement

The subjects' systolic and diastolic blood pressure will be measured twice with a 15-min interval in the left arm using a mercury barometer in a sitting position after a 10-min rest. Subjects will be asked to avoid smoking, drinking coffee, or heavy exercise for 30 min before the measurement. The average of the two measurements will be used to figure out the blood pressure.

### Biochemical assessment

Venous blood samples (10 cc) will be collected after 10 –12 h of fasting at the beginning and end of the study. The serum samples will be separated from whole blood (9 cc) by centrifugation at 3500 rpm for 10 min. The remaining 1 cc of whole blood samples will be stabilized in EDTA tubes. Both serum and whole blood samples will be immediately transferred to the freezer at -70 °C until the tests are performed. Serum TG and HDL-C concentrations will be measured using standard kits and enzymatic methods. Plasma atherogenic index (AIP) will be calculated through the formula AIP = logTG/HDL. Circulatory ox-LDL will be measured using the ELISA method and the relevant commercial kit using the autoanalyzer. Serum concentrations of Apolipoprotein A1 (ApoA1) and Apolipoprotein B (ApoB) values ​​will be assessed using the immunoturbidometry method. The concentration of serum malondialdehyde (MDA) will be determined based on the reaction with thiobarbituric acid (TBA) and using the spectrophotometric method. The serum values ​​of Total Antioxidant Capacity (TAC), Total Oxidant Status (TOS), Superoxide Dismutase (SOD), and Glutathione Peroxidase (GPx) will be measured using commercial kits and by spectrophotometric method. FBS will be measured on the same day of sampling using the glucose oxidase enzyme method. Insulin concentration will be measured by the ELISA method. Homeostatic Model Assessment for Insulin Resistance (HOMA-IR) will be calculated through the following formula: HOMA-IR = (FBS (mg/dl) x fasting insulin (μu/ml))/405.

### Adverse event reporting

The research executive team will be informed of any adverse side effects and will keep a record of any adverse incidents.

### Patient and public involvement

Patients or the public were not involved in the design, conduct, reporting, or dissemination plans of this research.

### Statistical methods

Data will be analyzed using SPSS software version 23. The normality of the distribution of the variables will be checked by the Kolmogorov–Smirnov test. All normal distributed quantitative data will be reported as mean ± standard deviation, and qualitative data will be reported as frequency (percentage). Analyses of non-normal variables will be performed after logarithmic transformation. Comparisons of baseline characteristics will be performed using independent t-test (for quantitative data) and chi-squared test (for qualitative data). Analysis of covariance (ANCOVA) test will be used to compare the effect of the intervention between the two groups by adjusting baseline values and confounding variables. A paired t-test will be used for within group comparisons. P < 0.05 will be considered to be statistically significant.

## Discussion

In recent years, the prevalence of MetS has significantly increased [23]. The pathophysiology of MetS is significantly influenced by oxidative stress, inflammation, and insulin resistance [24]. Studies show that dysbiosis may play a major role in the development and progression of these variables [25]. Probiotics can alter the gut bacteria to reduce the risk factors for metabolic syndrome [26]. The number of research examining the benefits of probiotic supplements is substantial, however there are few studies examining the effects of probiotic and prebiotic food products in metabolic diseases. However, no study has been conducted to evaluate the effect of synbiotic yogurt containing Lactobacillus plantarum, Lactobacillus pentosus, and Chloromyces marcosinos yeast in people with metabolic syndrome. In the present study, our aim is to investigate the effect of consumption of newly developed synbiotic yogurt containing Lactobacillus plantarum, Lactobacillus pentosus, and Chloromyces marcosianos on metabolic components, oxidative stress status, and some other cardiovascular disease risk factors in adults with MetS. If the appropriate impact of synbiotic yogurts on the outcome variables is identified in the current study, including these items in the dietary management of patients with MetS will be a cost-effective method of using probiotics.

## Data Availability

Not applicable.

## Abbreviations

MetS: Metabolic syndrome
NCEP: National Cholesterol Education Program
ROS: Reactive oxygen species
LDL: Low-density lipoprotein
Ox-LDL: Oxidized low-density lipoprotein
NO: Nitric oxide
MDA: Malondialdehyde
BMI: Body mass index
WC: Waist circumference
TG: Triglyceride
HDL: High-density lipoprotein
FBS: Fasting blood sugar
IPAQ: International Physical Activity Questionnaire
AIP: Plasma atherogenic index
ApoA1: Apolipoprotein A1
ApoB: Apolipoprotein B
TBA: Thiobarbituric acid
TAC: Total Antioxidant Capacity
TOS: Total Oxidant Status
SOD: Superoxide Dismutase
GPx: Glutathione Peroxidase
HOMA-IR: Homeostatic Model Assessment for Insulin Resistance
